# A Vision of State Medical Service

**Published:** 1919-01-18

**Authors:** L. L. Phillips

**Affiliations:** Brighton; T./Capt., R.A.M.C.


### THE EDITOR'S LETTER-BOX.

[Correspondence on all subjects is invited, but we cannot in any way be responsible for the opinions expressed by our correspondents, who must give their name and address as a guarantee of good faith, but not necessarily for publication. Correspondents are reminded that brevity of style and conciseness of statement greatly facilitate early insertion.]

### A VISION OF STATE MEDICAL SERVICE.

*To the Editor of* THE HOSPITAL.

SIR,—On reading Colonel Maurice's "Vision of a State Medical Service," one cannot but be struck with the trouble and pains he has taken to elucidate and place on a sound basis a scheme any form of which is bound to more or less revolutionise the Medical Profession. As Colonel Maurice rightly says, a State medical service, in some form or another, is certainly coming; of that a good many of us who were general practitioners in pre-war days are quite convinced. The Government are already pledged to drastic public health reconstruction, and the old form of private practice combined with medical officer of health, with its local and petty jealousies and lack of keeping up to date, by no means approaches the ideal which the war has taught us to aim at; therefore there is bound to be some form of State interference. Well, do, for goodness' sake, for once in a way, let the medical profession be united and prepared. Let us hope that the general practitioner element of the profession will, and will be allowed to, discuss with the Parliamentary authorities the form that these drastic reconstruction measures shall take. The B.M.A. and such-like formations have done good work in the past, no doubt, but they are too Parliamentary and not sufficiently *au fait* with the views of the average general practitioner. This time do not let us be let down by the impecunious minority and forced to take whatever scheme is put forward by a more or less lay committee, as was the case with the Insurance Act. Colonel Maurice's scheme, based on the model of the Army Medical Service, undoubtedly possesses great possibilities; but first and foremost there must be greater elasticity and more or less absence of the *absolutely* hidebound rules between the practising doctor and the district or higher officials; there must also be nothing like the delay and general loss of time that takes place in the Army Service. For instance, the telephone could be recognised as the official means of communication; delay in obtaining drugs or instruments would be fatal to smooth working; every district officer should be allowed a free hand up to quite a large sum of money. Thomas Atkins puts up with delay owing to discipline, but the private patient will not do so. The scheme must be worked on commercial business-like principles; miles of forms in duplicate and triplicate will never allow the scheme to work. Colonel Maurice says: "In a small country town there would be established a senior medical man, and with him juniors of various degrees of seniority . . . the sanitation of the town would be under their supervision." Surely it would be advisable to have the purely public health work entirely separated from the branch that treated the patients; each county or county borough director should have his entirely separate sanitary staff. Then with regard to hospitals. At present there is quite insufficient accommodation for the poor people, let alone the hospital accommodation for the entire population. The mere building of hospital accommodation is alone a gigantic scheme, though no doubt various hutments and war hospitals could be utilised for a time. Then as to how the profession could be reconciled, there is absolutely only one way: a special Act of Parliament must bring in at the commencement absolutely every man practising or about to practise, and the general success of the scheme in the course of years would reconcile the profession. If the Government took the bull by the horns and undertook the complete and total scheme from the commencement, I believe that the profession would adapt itself willingly. But, of course, men already in practice, and who have sunk capital in the purchase of those practices, would have to be recompensed. Of course, as the medical fledgling increased in numbers and had not sunk money in the purchase of a practice, that item of expenditure would gradually not appear; nor need all this money for recompense be found at once; probably a large part of it could take the form of increased pensions for those who had to be recompensed, as against the smaller pensions earned by those men entering the Service direct on qualification.

Colonel Maurice talks of the rewards offered to the other branches of the Civil Service as inducements. Surely the average practitioner would care little for the possibility of receiving recognition in the form of being able to wear a small piece of coloured ribbon, especially at the present time. Under Colonel Maurice's heading of "Earnings" the man most affected is the world-known consultant. Even though the scheme was started with the idea of the higher posts being real plums, these posts could hardly maintain his present financial position. Possibly the few would sacrifice themselves for the good of the majority, and in the course of time the coming men for such positions, if they were given posts such as charge of the medical or surgical divisions in a county or large town, would with their pay and special earnings (even though half were given to the State) be able to make sufficient to induce the best men to go into that branch of the profession. The smaller men could, as Colonel Maurice says, be graded to earn about what they are now earning, and with the prospect of a pension at their retirement, instead of the sale of their practice; but that pension would have to be a real retirement wage, at the lowest probably of a thousand pounds per annum.

Colonel Maurice says there need be no compulsion about men joining the scheme, but I am convinced that if anything is to be made of the scheme absolute compulsion is necessary to start with. On the whole, I consider that the scheme as worked out by Colonel Maurice is workable and good, but provided always that it is worked out on the understanding that it is to be an expensive, well-paid scheme (to really raise the standard of the profession), and that the rules are so framed that there is elasticity and, above all, no red tape delays. Should this scheme prove to be the future form of the medical profession in England, our thanks will be due to Colonel Maurice for his work and his desire to promote a band of well-paid comrades, of good social position, working together for the benefit of the common good and the nation at large.—Yours truly,

L. L. PHILLIPS,

Brighton, January 9, 1919. T./Capt., R.A.M.C.

☛ Articles and correspondence on this subject appeared in our issues of November 9, 16, 23 and 30, pp. 104, 109, 133, 169, 177, 178, and 187, December 7 and 14, pp. 197 and 229.

